# Impact of COVID-19 on the Mental Health and Well-Being of Latinx Caregivers of Children with Intellectual and Developmental Disabilities

**DOI:** 10.3390/ijerph18157971

**Published:** 2021-07-28

**Authors:** Yolanda Suarez-Balcazar, Mansha Mirza, Vanessa L. Errisuriz, Weiwen Zeng, Jasmine P. Brown, Sandra Vanegas, Nazanin Heydarian, Deborah Parra-Medina, Paula Morales, Hilda Torres, Sandy Magaña

**Affiliations:** 1 Department of Occupational Therapy, University of Illinois Chicago, 1919 West Taylor, Chicago, IL 60612, USA; mmirza2@uic.edu (M.M.); jpbrown2@uic.edu (J.P.B.); 2 Latino Research Institute, University of Texas at Austin, 210 W. 24th St., Austin, TX 78712, USA; vlerrisuriz@austin.utexas.edu (V.L.E.); parramedina@austin.utexas.edu (D.P.-M.); 3 Steve Hicks School of Social Work, University of Texas at Austin, 1925 San Jacinto Blvd., Austin, TX 78712, USA; weiwenzeng@utexas.edu (W.Z.); nmheydarian@miners.utep.edu (N.H.); mapaulamorales13@utexas.edu (P.M.); hildatorres98@live.com (H.T.); smagana@austin.utexas.edu (S.M.); 4 School of Social Work, Texas State University, Encino Hall, 712 North Commanche St., San Marcos, TX 78666, USA; svanegas@txstate.edu

**Keywords:** COVID-19, disability, Latinx, mental health, well-being

## Abstract

The COVID-19 pandemic has impacted the entire world in unprecedented ways. However, populations that have had a history of marginalization have experienced a more profound impact. One such group is Latinx families of children with intellectual and developmental disabilities (IDD) in the Unites States. In this study, we used a mixed methods approach to explore the impact of the pandemic on the mental health and well-being of Latinx caregivers of children with IDD. Specifically, we (1) identified which social determinants of health are correlated with maternal caregivers perceived general health, mental health, and well-being; (2) explored the impact of the pandemic on families’ overall eating and physical activity routines; and (3) identified emergent themes from caregivers’ experiences during the pandemic. Thirty-seven Latinx caregivers participated in three interviews in which several validated instruments were administered. The results indicated that perceived social support, annual family income, food security, and receipt of financial benefits were correlated with fewer depressive symptoms. Annual family income was also significantly correlated with perceived general health. Most caregivers reported that the pandemic had placed a strain on their economic situation; increased their isolation; and disrupted their child’s therapeutic supports, online education, eating routines, and engagement in physical activity. Meanwhile, some caregivers reported positive changes as a result of the pandemic. Implications for future research and practice are discussed.

## 1. Introduction

The COVID-19 pandemic dramatically impacted the physical, mental, economic, and social well-being of millions of people across the world. Furthermore, the pandemic exacerbated disparities already experienced by populations with a long history of marginalization. In the United States, Latinx communities, in particular, have experienced devastating health and economic consequences because of the pandemic. For instance, Latinxs have disproportionally higher COVID-19 incidence and death rates as compared with non-Latinx Whites [1,2,3]. Latinxs, many of whom are considered to be essential workers, are more likely to be exposed to COVID-19 and less likely to seek medical care when infected with the virus. This is, in part, because Latinxs are more likely to be uninsured as compared with other racial and ethnic groups [4]. The impact of COVID-19 goes beyond physical health, as many Latinx workers, particularly women, have been laid off or seen their work hours reduced [5]. All these factors, in turn, have contributed to higher levels of anxiety and stress among Latinx families.

The emphasis on the social and economic conditions created by the pandemic underscores the social determinants of health (SDOH) model, whereby social and economic conditions are at the root of health disparities [6,7,8]. The SDOH model calls attention to contextual factors including living conditions and environments that can inform social policies and systemic-level changes [9,10]. Social conditions, which are fundamental causes of health disparities [10], are related to race and ethnicity, social class, social networks, and social capital, as well as economic, and environmental factors.

The World Health Organization has emphasized the psychological impact of global diseases and pandemics as people cope with the restrictions imposed, and with the economic, physical, and mental health impacts [11]. During disasters, dramatic changes in everyday life, in addition to losing needed in-person supports and services lead to increases in anxiety, depression, and social isolation [12].

The COVID-19 pandemic has exacerbated the existing disparities experienced by some groups which are often ignored in health disparities discourses. One such group is Latinx caregivers of children with intellectual and developmental disabilities (IDD). Studies have indicated that disasters are more likely to have psychological and mental health consequences on adult caregivers as compared with those who are not caregivers [13]. A study conducted by Davidson et al. found significant levels of pandemic stress associated with worse mental health symptoms and financial outcomes among caregivers of young children [14]. Participants in the study reported anger, anxiety, sleep disturbances, social conflict, and preoccupations with changes in employment condition, housing, finances, personal and family health, and stress about fulfilling their caregiving responsibilities. Although the Davidson et al. (2020) study was conducted with caregivers of young children without disabilities [14], vast number of studies support that those caregivers of children with IDD experience additional challenges and stressors (e.g., [15]). Some studies on the impact of the pandemic on caregivers are emerging, however, little is known about the mental health and well-being of Latinx caregivers of children with IDD.

### 1.1. Latinx Caregivers of Children with Intellectual and Developmental Disabilities

Strong evidence indicates that caregivers of children with IDD experience stress due to the additional demands associated with raising a child with IDD (see [16,17]). This is particularly true for Latina caregivers who are more likely than other racial and ethnic groups to be the main caregiver of an entire family that includes children with IDD, other non-disabled children, and older parents. In addition, Latinx children with IDD may experience higher rates of obesity [18] and lower quality of healthcare as compared with White children with IDD [19]. All these issues compound to place additional demands on Latinx caregivers increasing their levels of stress and anxiety [20,21].

In previous studies, Latinx caregivers of children with IDD reported a lack of knowledge about community resources available to them and their children [22]. Latinx caregivers reported a lack of access to mental health resources in the community, relying instead on faith-based settings and each other for mental health support [23]. Caregivers also reported poor availability of quality healthcare [19,24] and limited access to culturally relevant programming that supports their health and well-being [25,26]. Overall, the evidence indicates that Latinx families of children with IDD were underserved even prior to the pandemic [27].

### 1.2. Latinx Caregivers of Children with IDD in Times of the COVID-19 Pandemic

The pandemic is impacting children with disabilities more drastically as compared with children without disabilities [28,29]. According to the American Association of Intellectual and Developmental Disabilities [12], caregivers of children with IDD are reporting higher levels of anxiety and depression relative to pre-pandemic and caregivers of children without IDD. One of the many reasons is the transition to online education and virtual therapeutic supports which have introduced new challenges for families resulting in additional stressors, particularly among the main caregivers. Such challenges include the loss of routines, gaps in online educational instruction, and lack of access to medical services. Children with disabilities from low-resource backgrounds (who are often from racial and ethnic minority groups) were more negatively impacted than affluent children due to the lack of access to laptops/computers and reliable internet connectivity at home, lower internet literacy, competing demands of other children, limited economic resources, high density living conditions, and lack of therapeutic supports [28,29].

Caregivers have also reported limited ability to cope with life stressors and increased social isolation [30]. Since most support services suspended face-to-face delivery, increasing reliance on family members, particularly the mother, is likely decreasing what limited coping capabilities were present prior to the pandemic. Strong evidence supports the relationship between social isolation, lack of social capital, and mental health concerns [31,32].

Overall, the restrictions in place due to the pandemic such as social distancing and lockdown policies, online education for children, job losses, and other changes have placed additional stressors on caregivers. A study conducted by Dhiman et al. in India reported a significantly higher prevalence of depression, anxiety, and stress symptoms among caregivers of children with IDD after the COVID-19 outbreak [30]. Similarly, Alhuzimi reported higher levels of stress among caregivers of children with autism in Saudi Arabia due to the pandemic [15].

Nygren and Lulinski reported that 74% of people with IDD had lost at least one or more services entirely, that only a small percentage of services were being delivered virtually, and not all supports were delivered successfully through the virtual interaction [12]. Children with IDD who often need supports at school and at home are experiencing more dramatic consequences.

There are limited studies on the impact of the pandemic on Latinx caregivers of children with IDD, particularlyon their general health, mental health, and well-being. More studies are needed to develop strategies to combat the pandemic’s negative impact on Latinx caregivers of children with IDD. Therefore, the overall aim of this study is to explore the impacts of the pandemic on the perceived general health, mental health, and well-being of Latinx caregivers. Specific research questions include: (1) Which social determinants are correlated with the perceived general health, mental health, and well-being of Latinx caregivers? (2) What are the impacts of the COVID-19 pandemic on these families’ daily routines including their eating and physical activity routines? and (3) What are the main themes emerging from caregivers’ experiences during the pandemic?

## 2. Materials and Methods

### 2.1. Study Design

Data for the current study were gathered as part of a larger cross-sectional research project that explored health behaviors (e.g., physical activity and diet) and lifestyle factors (e.g., home environment) that contribute to the high prevalence of overweight/obesity among Latinx children with IDD and their maternal caregivers. In the present study, we explored the impact of the pandemic on caregivers’ perceived general health, mental health, and well-being using a mixed methods analytic approach.

### 2.2. Participant Recruitment and Procedures

Researchers recruited maternal caregiver-child dyads from two urban cities in the United States. The study protocol was reviewed and approved by the Institutional Review Boards of the universities at both sites. Caregiver-child dyads were recruited through community agencies that serve the target population and shared recruitment materials (an informational flyer and an accompanying verbal recruitment script, both in Spanish and English) with Latinx families. The researchers created a Community Advisory Board (CAB) at each site to assist with recruitment and provide ongoing input to the research team. The CAB members were directors of local Latinx-serving agencies and/or parents of a child with an IDD, or a person with a disability themselves. Recruitment activities included posting the project’s bilingual brochure on community agencies’ websites, community outreach presentations about the project delivered by research staff, and social media advertisements on Facebook. Families expressed interest in the study by filling out an online study interest form. Research staff screened the families for eligibility prior to obtaining informed consent. At each site, the eligibility criteria included: (1) the caregiver identified as a mother (or other female primary caregiver who has custody of child) of Latinx descent; (2) the caregiver had a child with IDD between 6 and 17 years of age; (3) the focal child had a diagnosis of autism spectrum disorder, Down syndrome, and/or intellectual disability; and (4) the focal child was able to walk.

Data were collected in three separate phone or videocall interviews spread over 4–6 weeks, each interview lasting 60–75 min. Institutional restrictions were placed on in-person interviews at the time of data collection due to the pandemic. Between July 2020 and March 2021, thirty-seven caregiver-child dyads completed all three interviews, conducted by trained bilingual research staff. Research staff obtained verbal consent from the caregiver, parental consent for child participation, and child assent from the child(ren). During the first interview, research staff administered questionnaires regarding demographics and child health, health behaviors, quality of life, and home environment. At the second interview, caregivers responded to questionnaires about their own health, health behaviors, and well-being. At the third interview, research staff asked about physical activity and behaviors of both the caregiver and child as well as questions related to the impact of the pandemic on the family in general and on health behaviors specifically. Families had the option to complete assessments in English or Spanish. Participants received an incentive of a 25 USD gift certificate for each interview, with a total of 75 USD per family.

Regarding the participants, Table 1 shows demographic characteristics of the 37 caregiver-child dyads. On average, caregivers were 44 years old, and mostly foreign-born (86%) with the majority born in Mexico (78%). Among the caregivers, only 22% reported that they were employed/self-employed and most (63%) reported an annual family income of 35,000 USD or less. Almost half of the caregivers said they did not have health insurance at the time of the assessment. Meanwhile, children had a mean age of 11.5 years, the majority were male, born in the USA, and had a primary diagnosis of autism spectrum disorder. Most children were reported to have public insurance such as Medicaid or the Child Health Insurance Program (CHIP), while 17% had private insurance.

### 2.3. Instruments

With respect to demographic information, questions for caregivers included age, country of origin, health condition, marital and employment statuses, annual household income, and insurance type. We also collected child demographics based on caregiver-reported data including child age, place of birth, gender, IDD diagnoses, and insurance type.

Caregiver health was measured by the Medical Outcomes Study Questionnaire Short Form 36 Health Survey (SF-36) [33]. The SF-36 is an indicator of overall health status and has eight subscales: energy/fatigue, vitality, physical functioning, bodily pain, general health perceptions, physical role functioning, social role functioning, and mental health. The SF-36 has been validated and widely used across diverse patient groups and is available in Spanish [34]. The current study utilized overall perceived health and energy/fatigue scores. Perceived health was measured by a single item that asked the respondents to rate their overall health on a scale from 1 (poor) to 5 (excellent). The energy/fatigue subscale has 4 items that measure the respondent’s level of energy over the last 4 weeks (e.g., “Did you have a lot of energy?”). Response categories range from 1 (all of the time) to 6 (none of the time). The individual item scores were recoded into two indices of perceived health and energy/fatigue, according to the original scoring protocol [35]. The indices both ranged from 0 to 100 with higher scores indicating more favorable health states. Reliability of the energy/fatigue subscale in the present sample was good (α = 0.87).

Caregiver mental health was measured by the 20-item Center for Epidemiological Studies Depression Scale (CES-D) [36]. The CES-D assesses self-rated frequencies of depressive symptoms over the last week. Examples of symptoms include restless sleep and loss of appetite. Responses could range from 0 (rarely (less than 1 day a week)) to 3 (most of the time (5–7 days a week)). Individual item scores were summed to create a total CES-D score for each caregiver. Higher scores indicated greater depressive symptoms with a cutoff score of 16 or above used to identify those at risk for clinical depression [37]. The CES-D has been translated into Spanish and established as a valid and reliable measure of depressive symptoms in different racial and ethnic groups including Latinx populations [38,39,40]. Reliability of the CES-D was good for the present sample (α = 0.83).

Perceived social support was measured by the 12-item Multidimensional Scale of Perceived Social Support (MSPSS) [41]. The MSPSS measures availability of social support from family, friends, and a significant other (see https://gzimet.wixsite.com/mspss, accessed on 17 April 2021). The Spanish version has been validated and was found to have good internal reliability across subject groups [42]. Previous studies have demonstrated that the MSPSS has strong factorial, convergent, and divergent validity, as well as high internal consistency and test-retest reliability, among Latinx populations [43]. Each item is rated on a 7-point Likert scale ranging from 1 (very strongly disagree) to 7 (very strongly agree). Individual ratings were summed, and then divided by the number of items for an overall score. Higher scores indicated greater levels of perceived social support. Reliability of the MSPSS in the present sample was excellent (α = 0.96).

Questions related to the COVID-19 pandemic were included in the interview protocol to assess how the pandemic had affected families through its influences on key social determinants of health. Interviewers asked participants if: (1) any family members experienced negative economic changes (i.e., loss of employment, working less hours, and inability to find a job); (2) the family received any direct financial benefits (i.e., government-issued relief checks, unemployment benefits, small business support, and student loans relief); and (3) the child had access to services during the COVID-19 pandemic (i.e., online special education, routine therapies, and IDD-related services). To further analyze the impact of the pandemic on family daily life and routines, we inquired about changes experienced in the following categories: eating routine, eating habits, physical activity/exercise routine, sedentary time, and food access. If the maternal caregiver indicated “yes” to any of these categories, they were prompted to openly elaborate on the specific changes. Their responses were recorded directly in Research Electronic Data Capture (REDCap 10.3.5, Vanderbilt University, USA) in the source language and analyzed separately (see qualitative analyses).

Food security was assessed using the 6-item short form in the U.S.A. Household Food Security Survey Module from the United States Department of Agriculture (see https://snaped.fns.usda.gov/library/materials/us-household-food-security-survey-module, accessed on 17 April 2021). The food security scale assesses food eaten in a household in the past 12 months and whether the participant was able to afford the food they needed. As per United States Department of Agriculture (USDA) guidelines, a raw score ranging from 0 to 6 was calculated by summing the affirmatives across each of the six questions. Food security was classified as 0 to 1 (high or marginal food security), 2 to 4 (low food security), or 5 to 6 (very low food security).

Moderate-to-vigorous physical activity (MVPA) was measured using the Community Health Activities Model Program for Seniors (CHAMPS) physical activity questionnaire [44]. The CHAMPS questionnaire is a 41-item self-report measure of physical activity that was developed originally for older adults but has been used with the general adult population and racially and ethnically diverse populations [44,45,46]. The CHAMPS questionnaire asks participants to recall their physical activity over the past month by reporting the typical number of hours per week that they engage in specific activities (e.g., dance, heavy gardening, yoga, walking, etc.). We modified age-specific items to be more age neutral (e.g., replacing “visit a senior center” with “visit a community center”). Responses were used to calculate time (hours) spent engaging in MVPA over the past week.

### 2.4. Data Analysis

#### 2.4.1. Quantitative Analyses 

Quantitative analyses were conducted using IBM SPSS version 24 (Armonk, NY, USA). Descriptive statistics for caregivers and child demographic characteristics, and economic (i.e., negative economic changes and receipt of financial benefits), social (i.e., child service access, food security, and perceived social support), and health-related variables (i.e., leisure-time MVPA, perceived health, energy/fatigue, and CES-D) during the COVID-19 pandemic were calculated. Then, we ran bivariate correlational analyses for continuous variables. We also ran independent samples *t*-tests using dichotomous demographic (caregiver insurance status and annual family income) and social determinants (food security, negative economic change, and receipt of financial benefits) factors as independent/grouping variables, and continuous outcomes (perceived health, energy/fatigue, and CES-D scores) as dependent variables. Finally, we mapped these correlates with our qualitative data to further illustrate how the COVID-19 pandemic affected the family’s routines, and a caregiver’s health, mental health, and well-being.

#### 2.4.2. Qualitative Analyses

A team-based analytic approach was used to examine responses to the open-ended questions. Four members of the research team (two monolingual and two bilingual native Spanish speakers) were involved in coding. The responses to open-ended questions were abstracted from REDCap for qualitative analyses. The responses were typed into REDCap in real time by research staff who were instructed to capture responses verbatim in the language (Spanish or English) used by the participant. For data transcription and coding, first, one of the bilingual coders translated all Spanish responses to English. Next, the second bilingual coder reviewed the translation for accuracy followed by a meeting between the two bilingual coders to resolve disagreements. Once all data had been translated to English, all four coders independently reviewed the data followed by a team meeting where emerging insights were discussed. A decision was made to use a deductive analytic approach [47], whereby, data were categorized into themes based on interview questions. A codebook including titles and descriptions for codes and subcodes was developed. All four coders conducted two rounds of independent coding. Team meetings were held before and after each round to discuss discrepancies and refine the codebook (e.g., adding new subcodes and merging codes) as needed. Coding disagreements were resolved through discussion and consensus, and where necessary, responses communicated in Spanish were re-reviewed to ensure that nuances were not lost in translation. After the codes were reconciled, they were organized into thematic categories by one of the coders. Thematic categories and the relationships between them were presented to the research team and revised based on feedback obtained. ATLAS.ti version 8.0 (ATLAS.ti Scientific Software Development GmbH, Berlin, Germany) was used to facilitate organization of codes and themes.

## 3. Results

### 3.1. Quantitative Results

With respect to social determinants of health, 38% of families reported having low or very low food security during the COVID-19 pandemic. Most families (70%) experienced at least one negative economic change and the majority (62%) did not receive any financial benefits from the government. More than half of the children (54%) were able to maintain access to two of the three service categories (i.e., online special education, routine therapies, and IDD-related services), while only 19% had access to all three (see Table 2).

#### Caregiver Physical Activity, Perceived Social Support, Health, and Mental Health

Table 3 shows the descriptive statistics of the standard measures used in this study. On average, caregivers reported: (1) spending 4.9 h per week on leisure-time MVPA; (2) moderately high perceived social support (*M_MSPSS_* = 5.37), with caregivers reporting greatest support from a significant other (*M_S_* = 5.65); (3) “good” (46%) or “fair” (32%) perceived health and a moderate level of energy/fatigue (*M_E/F_* = 52.0; (higher scores indicated greater levels of energy); and (4) moderately high overall depressive symptoms (*M_CES-D_* = 12.2), with 36% at risk of having depression (CES-D scores ≥16).

Bivariate correlations between continuous predictors (MSPSS and total numbers of child service access) and outcome variables (i.e., perceived health, energy/fatigue, and CES-D) revealed significant correlations (see Table 4). Greater perceived social support was significantly correlated with greater energy/fatigue scores and lower depressive symptoms scores. The total number of child services accessed was not correlated with any of the outcome variables. Within the outcome variables, greater perceived health and energy/fatigue scores were significantly correlated with lower depressive symptom scores.

Table 5 shows the independent samples *t*-test statistics. For perceived health, caregivers who had an annual family income of $35,001 USD or more reported significantly higher perceived health scores than those whose annual family income was 35,000 USD or less (*t* = 2.39, *p* = 0.02). For energy/fatigue scores, receipt of any financial benefits was significantly correlated with greater levels of energy among the caregivers (*t* = 2.61, *p* = 0.01). For depressive symptoms (CES-D) scores, higher level of annual family income (*t* = −3.60, *p* < 0.001), food security (*t* = −2.28, *p* = 0.03), and receipt of any financial benefits (*t* = −3.21, *p* = 0.003) were significantly correlated with lower depressive symptoms scores.

### 3.2. Qualitative Results

**Figure 1:**
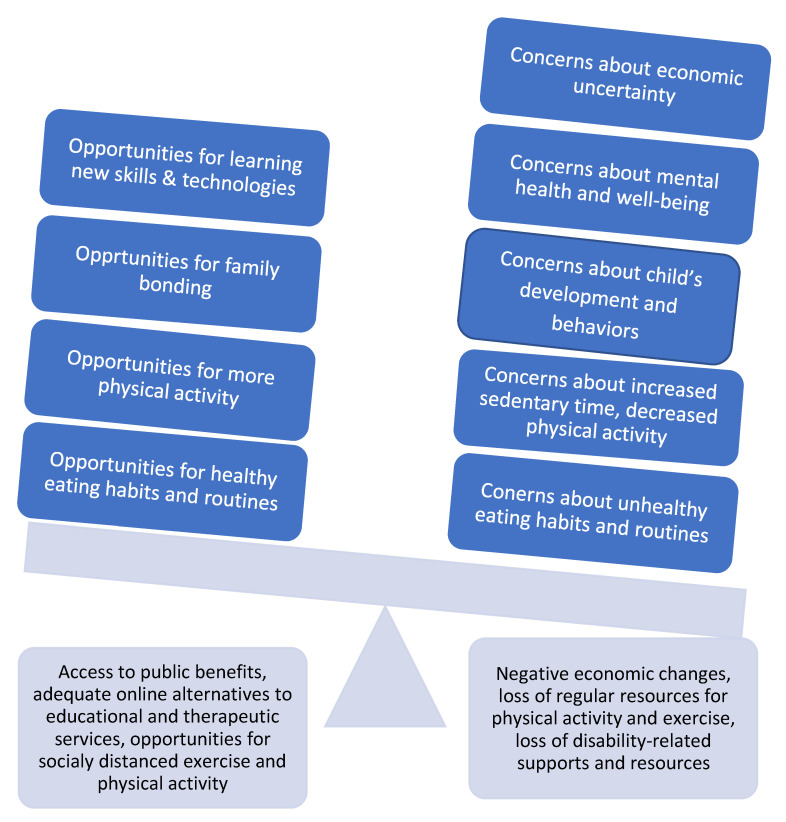
Themes and the dynamic relationship between them.

The qualitative results are presented across the following areas impacted by the COVID-19 pandemic: economic impact, mental health and well-being, child’s development and behavior, physical activity, and eating habits and routines. The qualitative data revealed that the pandemic presented both concerns and challenges, as well as opportunities for families. Families’ abilities to navigate challenges and make the most of opportunities appeared to be related to their access to community resources and supports. Figure 1 depicts the main themes and the relationships between them.

#### 3.2.1. Economic Impact

Economic uncertainty was an important concern reported by caregivers. In addition to concerns about the general economy, the pandemic also had a negative impact on family finances. As reported in Table 2, twenty-six caregivers had experienced at least one negative economic change, such as one or more family earners losing their job or working fewer hours. While 14 families had received financial benefits, such as stimulus checks (coronavirus economic impact payments) from the United States federal government, three caregivers reported their families were ineligible for these payments owing to their immigration status, one family lost the check that was issued, and one was anticipating a check, but it never arrived. For one family, negative economic changes were compounded because they had lost their public benefits and were unsure about the renewal process during the pandemic-induced lockdown.

One caregiver (35-year-old mother of an 8-year-old child with autism) said, “Had [benefit] before but it expired and now we don’t know how to renew it since the interview to renew it was in-person”. For families that were able to access public benefits and other community resources such as food pantries, these sources of support appeared to mitigate to some extent the economic challenges presented by the pandemic. As stated by one caregiver (45-year-old mother of a 12-year-old child with autism, intellectual disability, and sleep disorder), “Our community has really stepped up to help people who are struggling, and we have benefitted from that as well. We have received food at no cost, tests at no cost, and other things like clothes, books, Christmas gifts”.

#### 3.2.2. Mental Health and Well-Being 

Caregivers discussed how the pandemic had affected their mental health and well-being as well as the mental health of their children. Common stressors for caregivers included concerns about family finances and sustained employment, lack of support with childcare, and worries about family members getting sick. One mother (43-year-old mother of a 15-year-old child with autism) said, “I am a single mother, I don’t [have] a sitter. Mentally it [the pandemic] has also affected me. I had three panic attacks. We went through the virus, I felt it more”. Another caregiver (40-year-old mother of a 17-year-old child with autism) said, “It is easy to worry about the family’s health, especially since my dad has diabetes. Even though we already had the coronavirus, we can still get sick, it is hard and makes me worry, we try to be careful, but we don’t know where we could’ve gotten sick”. 

Concerns about children’s mental health mostly stemmed from lack of social interaction due to the pandemic lockdown. Eight caregivers felt that being at home all the time with limited contacts with peers was negatively affecting their child’s sociability and mental health. As one caregiver (35-year-old mother of an 8-year-old child with autism) said, “… due to the amount of time spent inside because [children] don’t have contact with other people or a friend group… being away from others is having a great impact, the kids aren’t sociable”. Another caregiver (45-year-old mother of an 11-year-old child with intellectual disability and Down syndrome) replied, “… my daughter was receiving…services before for mental health and now, they are cancelled. I am worried about the limited social interactions the girls [child with a disability and her sibling] are having and it has had a negative impact on their progress. My daughters are very anxious because they don’t understand why we can’t go outside anymore”. One caregiver (45-year-old mother of a 12-year-old child with autism, intellectual disability, and sleep disorder) discussed how staying at home all day had exacerbated her son’s social challenges. Her son had become more scared to leave the house and required a lot of effort to be convinced to go outside. In her words, “I am very concerned about my son’s mental health, not being able to go to school in person, and having all extracurriculars away has had a huge toll on his mental health. We even had to hospitalize him for 10 days because he was so dysregulated, and this continues to be my most pressing concern”. 

There were some families, however, that adapted to the pandemic-induced changes in their routines and were making the best use of their time together during lockdown. For twelve families, the pandemic had presented opportunities for bonding. One caregiver described how a stronger bond was developing between her children and their dad since he was forced to be home. Nine caregivers described spending more time together as a family, sharing meals and playing board games. For two families, children not having to go to school meant that mornings were less stressful, and children were able to sleep in and get more rest. These changes were perceived as positive and had led to an overall sense of enhanced well-being for these caregivers.

#### 3.2.3. Child’s Development and Behaviors

As schools and therapy sessions pivoted to an online format during pandemic lockdown, caregivers commented on the impact of this change on their children’s skill development and behaviors. At least 17 caregivers reported that educational and therapeutic services were curtailed or eliminated altogether. For example, one caregiver shared that her child’s school did not provide online speech and physical therapy services. This family could not afford these services outside the school system as they did not have health insurance. Two families had been offered therapeutic and educational services online but had difficulties accessing them due to unreliable computer and internet access. According to one caregiver (32-year-old mother of a 9-year-old child with autism and intellectual disability), “It took a long time (two months) to get internet access, and the children (all) lost those months of schooling, including special education”. The disruption of regular, face-to-face school and therapy sessions, and extracurricular activities made some caregivers concerned about their children’s social isolation, and the subsequent impacts on their developmental and academic skills.

While some families faced disruptions and difficulties with online services, other families saw benefits and opportunities. At least six families were satisfied with the online therapy services their child was receiving, which they perceived as either comparable to or even better than what they were receiving pre-pandemic. One caregiver (40-year-old mother of a 17-year-old child with autism) said, “[Son] has one therapy, at the beginning I thought it wasn’t going to work, but now he is more attentive and he doesn’t like missing the hour of therapy that he has. I think it is good”.

Five caregivers reported positive changes associated with online schooling such as exposure to learning apps and educational resources that had helped with acquiring academic and functional skills using technology. As one parent (45-year-old mother of a 12-year-old child with autism, intellectual disability, and sleep disorder) said, “We have been able to engage in remote learning with our son, our team has been very responsive and flexible, they have provided a whole set of teaching instruments that he usually uses in school for his use at home …”. One caregiver remarked that her daughter had developed new functional skills with using technology. Another caregiver expressed that with everyone being at home she had more time to work on personal hygiene, dressing and grooming skills with her daughter.

#### 3.2.4. Physical Activity

Twenty-three caregivers reported increased sedentary time and decreased physical activity for themselves and/or their children since the onset of the pandemic. Lack of access to regular physical activity outlets (e.g., gyms, recreation centers, public swimming pools, public parks, zoos, and libraries) was frequently cited as a reason for this change. As one caregiver (45-year-old mother of a 12-year-old child with autism, intellectual disability, and sleep disorder) said, “I am not going outside almost ever, and I spend a lot more time sitting down than I usually do because my routines in general have changed. For example, I used to walk to the library, but it’s been closed for months. It feels like you don’t have anywhere to go outside, so why go at all”. For children, the increase in sedentary time was further exacerbated by online schooling, loss of structured recess activities and physical education classes at school, and limited options for extracurricular activities outside of school. In the words of one caregiver (42-year-old mother of a 10-year-old child with autism), “[Son’s name] has been missing out on gym class, running around the gym and playing at recess. I (mom) have been missing my daily walks, walking to errands and Zumba classes”.

In addition, families living in small apartments were unable to find in-home alternatives for physical activity. One caregiver (39-year-old mother of a 10-year-old child with autism and intellectual disability) described the situation as follows, “[My] daughter is spending more time doing sedentary activities like playing on iPad, drawing, or swinging in home. Additionally, [our] apartment is small and limits the amount of movement the children can do”. Another caregiver (41-year-old mother of a 9-year-old child with autism) stated, “[Son] was in swimming lessons and practicing for the Basketball Special Olympics but he now doesn’t have anything”. In contrast, twelve caregivers reported spending more time pursuing physical activity and exercise. It appeared that these caregivers had the luxury of more free time during the pandemic and had access to hiking trails nearby, backyards, or larger homes that could accommodate indoor exercise equipment.

#### 3.2.5. Eating Habits and Routines

As with physical activity, 28 caregivers also reported changes in eating habits and routines. For some, the changes were positive, for others not so much. For example, with money being tight, eight families reported that they had to rely on food banks or other sources of food supply resulting in less control over the quality of food they consumed and having to make do with what was available. As one caregiver (55-year-old mother of a 17-year-old child with intellectual disability and Down syndrome) said, “It’s been hard because [we are] not able to afford a lot of food that is not canned”. Being unable to control ingredients and their quality posed special problems for caregivers looking after children with food sensitivities. As one caregiver (44-year-old mother of an 11-year-old child with autism and intellectual disability) stated, “Before, my daughter was gluten free and organic, eating a strict diet and now we can’t buy it”.

However, families reported divergent experiences. For example, one caregiver reported that they were able to afford basic groceries and used food pantries to supplement their diet with fresh fruits and vegetables. Another family was receiving food aid from their child’s school which included boxes of fruit, vegetables, juice, cereal, and milk, which they deemed satisfactory for their needs. For families that were able to afford quality foods, having children at home also meant being able to exercise more control over their nutritional intake. Four caregivers reported their family was making a conscious effort to eat more nutritious food and drink more water instead of sugary drinks. Nine caregivers also reported more home cooking, experimenting with new recipes, and putting more thought into preparing family meals. Being able to eat meals together as a family was also identified as a positive change, although for some, the temptation to eat more snacks while at home was a concern. As one caregiver (45-year-old mother of an 11-year-old child with intellectual disability and Down syndrome) said, “We do three meals together daily, we choose better foods, less sugary and salty, more vegetables, more fruit, and we are drinking more water instead of juices or sodas”. 

## 4. Discussion

To the best of our knowledge, this study is one of the first to explore the impact of the COVID-19 pandemic on the general health, mental health, well-being, and family routines of Latinx caregivers of children with IDD. Overall, the results indicated that more than one third of the Latinx caregivers were at risk of having depression, based on caregiver self-reported CES-D scores. Their perceived social support and having access to financial benefits were correlated with lower depressive symptom scores. Food insecurity was related to higher levels of depressive symptoms, while higher annual family income was both correlated with better perceived general health and lower levels of depressive symptoms. Most caregivers reported how the pandemic had placed strains on their economic situation, disrupted their child’s therapeutic supports, online education, eating routines, and engagement in physical activity. Nevertheless, some caregivers reported benefits they experienced as a result of the pandemic including more time spent with the family and stronger bonds with their children.

Grounded in the SDOH model, the results are consistent with current evidence indicating the negative impact of financial concerns on the levels of stress of parents [48] and the role of social support on overall well-being [14]. Strong evidence indicates that social support can mediate well-being and mental health outcomes [49].

Families of children with IDD face unique challenges because of their children’s disability, which have been exacerbated by the pandemic [15]. According to caregivers who participated in this study, the pandemic has had an impact on their own employment status, or that of a family member, their overall health and well-being, as well as their eating and physical activity routines. However, prior to this study, we knew little about the impact of the pandemic on Latinx families of children with IDD. Often children with IDD are overlooked when examining racial and ethnic disparities. Therefore, this study has multiple implications for policy, practice, and current research. Current policies offered by the United States federal government have included stimulus checks to support families through the economic upheaval caused by the pandemic. Our findings suggest the need for funding to augment culturally relevant support systems and mental health services, which are much needed by Latinx families of children with disabilities.

Although we did not specifically ask about immigration status, the fact that most of the caregivers are first generation immigrants may speak to the lack of knowledge of financial and food resources available to them, feeling disempowered to ask for support, or experiencing other barriers such as undocumented immigration status. Immigrant families may be hesitant to ask for financial and food supports due to fear of immigration practices and policies [50].

### 4.1. Community Efforts to Promote the Well-Being of Latinx Caregivers of Children with IDD

The research team has responded in different ways to support families of children with IDD. First, after completing the third interview, based on the information collected, the researchers created a personalized mini report to be shared with each family. The two-page report included a descriptive and graphic summary of the caregiver and child’s levels of physical activity, nutritional intake, and a list of resources and recommendations to support access to healthy foods and physical activity. Second, based on early data collected, we produced infographics to distribute to the community and other stakeholders. The infographics included highlights of the results and a list of resources in the community according to area of impact, i.e., financial resources, mental health support, food aids, and resources and settings for physical activity. These infographics are available in both English and Spanish and have been disseminated widely.

Third, the research team has optimized the partnerships created with community organizations serving Latinx families of children with disabilities to respond to the impact of the pandemic on families. With input from the Community Advisory Board members, we have organized webinars delivered by the researchers to Latinx families of children with disabilities. The webinars’ topics are based on needs of the community identified by the Community Advisory Board members who are themselves individuals with disabilities or parents of youth with disabilities, and/or directors of our community partner agencies. These webinars have met some of the needs of participants and have been well attended and well received. Topics have included promoting well-being in times of COVID, mental health support for children with disabilities and caregivers, advocating for the needs of their children in an online environment, protecting their rights, and disability and sexuality. Through these interactive webinars, parents have received useful information and support.

### 4.2. Limitations

The results from the current study need to be interpreted with caution due to some limitations noted here. First, our sample size significantly limited our ability to draw robust conclusions about the correlations between social determinants of health and caregiver health, mental health, and well-being. Due to the small sample, we were only able to conduct bivariate correlational analysis and independent samples *t*-tests, which did not allow us to adjust for other variables. For instance, our ability to detect whether food security and negative economic change are unique predictors of well-being outside of annual family income was hindered, as all the financial or economic-related variables were moderately correlated. Second, all standardized measures used in this study were based on caregiver self-report. For measures such as those for perceived social support, food security, and physical activity, data may have been subjected to social desirability bias (i.e., caregivers telling us what they think is socially appropriate/desirable). Third, while we used mixed methods to triangulate quantitative data with qualitative data, and thus gain more in-depth insights [51], we need to acknowledge that our findings may have limited generalizability to the larger population of Latinx families caring for a child with IDD in the USA. Furthermore, although the 37 caregiver-child dyads were interviewed in a period of 8 months, we anticipate that families may have been impacted differently across the duration of the pandemic. Future studies should include a larger sample of participants, refine analytic procedures, and conduct comparisons across time and sites, as well as consider measures of acculturation. Finally, future studies should explore how the experiences of Latinx families, during the pandemic, differ from that of other racial and ethnic minority families of children with disabilities and that of Latinx families of children without disabilities. Although evidence clearly supports that the pandemic has impacted everyone across the globe, the unique experiences of families of children with disabilities from diverse racial and ethnic backgrounds are worth exploring.

## 5. Conclusions

Limited research to date has studied the impact of the pandemic on caregivers of children with IDD and their families. Just as the general population has experienced changes in everyday life due to the pandemic, these changes might be more pronounced for Latinx families of children with IDD. Substantial disruptions in family finances and in educational and therapeutic services exacerbate the health disparities this population is already experiencing. The mental health of Latinx caregivers is also at risk, as most of the responsibility of caring for the child with IDD rest on the female caregiver. The levels of stress experienced by Latinx caregivers call for the need to leverage mental health and social supports for this population. There is a need for partnerships with community organizations to create support mechanisms, such as online and in-person groups and individual counseling, and to offer culturally and linguistically competent mental health services to Latinx caregivers and children with IDD.

## Figures and Tables

**Table 1:** Family/caregiver (*N* = 37) and child (*N* = 37) demographic characteristics.

Demographic	Family/Caregiver		Child
Characteristics			
	Mean (SD)		Mean (SD)
Age ^a^	43.9 (6.9)	Age	11.5 (3.6)
	***n* (%)**		***n* (%)**
US-born ^a^	5 (13.9)	US-born	34 (91.9)
Married/living with a partner^a^	28 (80.0)	Male	25 (67.6)
Insurance type ^a^		Insurance type ^a^	
None	17 (48.6)	None	1 (2.8)
Private	7 (20.0)	Private	6 (16.7)
Medicaid/Medicare	5 (14.3)	Medicaid/CHIP	27 (75.0)
Other	6 (17.1)	Other	2 (5.6)
Employment status ^a^		Primary diagnosis ^b^	
Unemployed/unable to work	15 (41.7)	ASD/Asperger	29 (78.4)
Employed/self-employed	8 (22.2)	ID/DS	7 (18.9)
Full-time caregiver	13 (36.1)	Sleep disorder	1 (2.7)
Annual family income ^a^			
35,000 USD or less	22 (62.9)		
35,001 USD or more	13 (37.1)		

Notes: ^a^ Sample sizes varied due to missing data as follows: family/caregiver age (*n* = 36), place of birth (*n* = 36), marital status (*n* = 35), insurance type (*n* = 35), employment status (*n* = 36), and annual family income (*n* = 35), child insurance type (*n* = 36). ^b^ Child diagnoses included: ASD, autism spectrum disorder; ID, intellectual disability; DS, Down syndrome. SD, standard deviation; CHIP, Child Health Insurance Program.

**Table 2:** Social determinants of health during the COVID-19 pandemic.

Social Determinants of Health	*n* (%)		*n* (%)
Food security ^a^		Child service access ^b^	
High or marginal	21 (61.8)	None	4 (10.8)
Low or very low	13 (38.2)	Access to 1 service	6 (16.2)
Negative economic change		Access to 2 services	20 (54.1)
None	11 (29.7)	Access to all 3 services	7 (18.9)
Yes, at least one	26 (70.3)		
Receipt of financial benefits			
None	23 (62.2)		
Yes, at least one	14 (37.8)		

Notes: ^a^ Sample sizes varied due to missing data: Food security (*n* = 34). ^b^ Categories of child service access: online special education, routine therapies, and IDD-related services.

**Table 3:** Caregivers’ physical activity, perceived social support, and standardized outcome measures and distributions.

Measures	Valid *n*	Mean	Median	SD	Skewness	Kurtosis	Min–Max
Leisure-time MVPA	36	4.9	4.0	4.8	1.0	0.44	0–17
MSPSS	36	5.4	5.5	1.2	−0.68	−0.01	2.25–7
Perceived health	36	41.0	50.0	21.7	0.24	0.65	0–100
Energy/fatigue	37	52.0	50.0	19.8	−0.40	−0.09	5–90
CES-D	36	12.2	12.0	7.6	0.17	−0.72	0–28

Notes: SD, standard deviation; MVPA, moderate to vigorous physical activity; MSPSS, multidimensional scale of perceived social support; CES-D, Center for Epidemiological Studies depression scale.

**Table 4:** Bivariate correlations between outcome and predictor variables.

	1	2	3	4	5
1. Perceived Health	-	0.16	−0.46 **	0.30	0.02
2. Energy/Fatigue (Higher score = greater energy)		-	−0.48 **	0.50 **	−0.06
3. CES-D total scores			-	−0.58 ***	−0.13
4. MSPSS total scores				-	0.14
5. Total numbers of child service access					-

Note: ** *p* < 0.01, and *** *p* < 0.001. CES-D, Center for Epidemiological Studies depression scale; MSPSS, multidimensional scale of perceived social support.

**Table 5:** Independent samples *t*-tests statistics.

Outcomes	Predictors	Reference ^a^ Mean (*n*)	Comparison ^b^ Mean (*n*)	*t*	df	*p*-Values	95% CI
Perceive health	Caregiver insurance status	35.3 (17)	47.2 (18)	1.66	33	0.11	[−2.7, 26.6]
	Annual family income	34.5 (21)	51.9 (13)	2.39	32	0.02 *	[2.5, 32.3]
	Food security	34.6 (13)	45.2 (21)	1.38	32	0.18	[−5.0, 26.3]
	Negative economic change	37.0 (25)	50.0 (11)	1.70	34	0.10	[−2.5, 28.5]
	Receipt of financial benefits	37.5 (22)	46.4 (14)	1.21	34	0.23	[−6.0, 23.9]
Energy/fatigue	Caregiver insurance status	49.4 (17)	56.7 (18)	1.09	33	0.29	[−6.3, 20.8]
	Annual family income	49.3 (22)	56.5 (13)	1.03	33	0.31	[−7.1, 21.6]
	Food security	46.9 (13)	57.6 (21)	1.55	32	0.13	[−3.3, 24.7]
	Negative economic change	49.8 (26)	57.3 (11)	1.05	35	0.30	[−7.0, 21.9]
	Receipt of financial benefits	45.9 (23)	62.1 (14)	2.61	35	0.01 *	[3.6, 28.9]
CES-D	Caregiver insurance status	13.8 (16)	10.8 (18)	−1.14	32	0.26	[−8.5, 2.4]
	Annual family income	15.0 (22)	6.4 (12)	−3.60	32	<0.001 ***	[−13.4, -3.7]
	Food security	15.8 (13)	10.0 (21)	−2.28	32	0.03 *	[−11.2, −0.6]
	Negative economic change	13.4 (25)	9.5 (11)	−1.45	34	0.16	[−9.4, 1.6]
	Receipt of financial benefits	15.0 (22)	7.6 (14)	−3.21	34	0.003 **	[−12.1, −2.7]

Notes: ^a^ Reference groups: caregiver not insured, annual family income ≤$35,000 USD, low or very low food security; experienced at least one negative economic change, received none of the financial benefits. ^b^ Comparison groups: caregiver insured (any type), annual family income >35,001 USD, high or marginal food security, did not experience any negative economic changes, received at least one financial benefit. * *p* < 0.05, ** *p* < 0.01, and *** *p* < 0.001. df, degrees of freedom; CI, confidence interval; CES-D, Center for Epidemiological Studies depression scale.

## Data Availability

The data presented in this study are available on request from the Principal Investigator: Sandra Magaña
